# Fenoldopam use in a burn intensive care unit: a retrospective study

**DOI:** 10.1186/1471-2253-10-9

**Published:** 2010-06-24

**Authors:** John W Simmons, Kevin K Chung, Evan M Renz, Christopher E White, Casey L Cotant, Molly A Tilley, Mark O Hardin, John A Jones, Lorne H Blackbourne, Steven E Wolf

**Affiliations:** 1 United States Army Institute of Surgical Research, 3400 Rawley E. Chambers Avenue, Fort Sam Houston, Texas, 78234, USA; 2 Wilford Hall Medical Center, 2200 Bergquist Drive, San Antonio, Texas, 78236, USA; 3 UT Health Science Center at San Antonio, 7703 Floyd Curl Drive, San Antonio, Texas, 78229, USA; 4 Uniformed Services University of the Health Sciences, 4301 Jones Bridge Road, Bethesda, Maryland, 20814, USA

## Abstract

**Background:**

Fenoldopam mesylate is a highly selective dopamine-1 receptor agonist approved for the treatment of hypertensive emergencies that may have a role at low doses in preserving renal function in those at high risk for or with acute kidney injury (AKI). There is no data on low-dose fenoldopam in the burn population. The purpose of our study was to describe our use of low-dose fenoldopam (0.03-0.09 μg/kg/min) infusion in critically ill burn patients with AKI.

**Methods:**

We performed a retrospective analysis of consecutive patients admitted to our burn intensive care unit (BICU) with severe burns from November 2005 through September 2008 who received low-dose fenoldopam. Data obtained included systolic blood pressure, serum creatinine, vasoactive medication use, urine output, and intravenous fluid. Patients on concomitant continuous renal replacement therapy were excluded. Modified inotrope score and vasopressor dependency index were calculated. One-way analysis of variance with repeated measures, Wilcoxson signed rank, and chi-square tests were used. Differences were deemed significant at p < 0.05.

**Results:**

Seventy-seven patients were treated with low-dose fenoldopam out of 758 BICU admissions (10%). Twenty (26%) were AKI network (AKIN) stage 1, 14 (18%) were AKIN stage 2, 42 (55%) were AKIN stage 3, and 1 (1%) was AKIN stage 0. Serum creatinine improved over the first 24 hours and continued to improve through 48 hours (*p *< 0.05). There was an increase in systolic blood pressure in the first 24 hours that was sustained through 48 hours after initiation of fenoldopam (*p *< 0.05). Urine output increased after initiation of fenoldopam without an increase in intravenous fluid requirement (*p *< 0.05; *p *= NS). Modified inotrope score and vasopressor dependency index both decreased over 48 hours (*p *< 0.0001; *p *= 0.0012).

**Conclusions:**

These findings suggest that renal function was preserved and that urine output improved without a decrease in systolic blood pressure, increase in vasoactive medication use, or an increase in resuscitation requirement in patients treated with low-dose fenoldopam. A randomized controlled trial is required to establish the efficacy of low-dose fenoldopam in critically ill burn patients with AKI.

## Background

Fenoldopam mesylate is a highly selective dopamine-1 receptor agonist approved for the treatment of hypertensive emergencies that may have a role at low doses in preserving renal function in those at high risk for or with acute kidney injury (AKI) [1]. Historically, non-selective dopaminergic stimulants have been of mixed benefit as the improvement in renal vascular resistance, glomerular filtration rate (GFR), and sodium excretion has been counterbalanced by hypotension and arrhythmias [2]. Because fenoldopam is a pure dopamine A-1 agonist, it allows a more targeted approach to manipulate renal hemodynamics while minimizing systemic symptoms [3]. Its renal effects include decreased renal vascular resistance, increased GFR, increased natriuresis through inhibition of the Na/H exchanger and Na/K/ATPase-mediated sodium reabsorption in the proximal tubules, and water diuresis via the inhibition of antidiuretic hormone (ADH). For treatment of AKI, fenoldopam is postulated to work by restoring renal blood flow (RBF) via non-nitric oxide mediated arterial dilation [4].

Fenoldopam was initially indicated for augmentation of RBF during treatment for hypertensive emergency. Its use is associated with a dose-dependent (up to 0.5 mcg/kg/min) antihypertensive effect and increased RBF [5,6].

Subsequent studies have attempted to exploit fenoldopam effect on RBF with varying degrees of success. The literature does not clearly indicate a reduction in contrast-induced nephropathy in cardiovascular surgery patients although most of the studies indicate either a decrease in renal failure or a minimization of AKI [7-15]. Some studies show a benefit primarily in non-diabetic patients, whereas others show the opposite [16,17]. Fenoldopam use is also being evaluated in the high-risk pediatric population as a diuretic and anti-hypertensive [18,19]. A recent meta-analysis suggests a reduction in the need for renal replacement therapy and mortality in patients with AKI and fenoldopam use [20].

AKI is associated with increased morbidity and mortality in medical, surgical, and BICU patients [21-27]. Additionally, aggressive treatment has been shown to improve mortality in the burn population [28,29].

Concern remains regarding fenoldopam's antihypertensive effect. Fenoldopam has been associated with hypotension, tachycardia, congestive heart failure, myocardial infarction, and hypokalemia. Care should be taken when considering using fenoldopam in patients on beta blockers and diuretics as this may increase the risk of hypotension and hypokalemia.

There are no data on fenoldopam use in the burn population. Therefore, the purpose of our study was to describe our use of low-dose fenoldopam (0.03-0.09 μg/kg/min) infusion in critically ill burn patients with AKI.

## Methods

A retrospective review of consecutive patients admitted to our BICU was approved by the local institutional review board. The database includes all patients admitted to the BICU with burns from November 2005 to September 2008 who received low-dose fenoldopam.

Initiation of fenoldopam infusion was at the discretion of the attending physician, but criteria typically used were low urine output despite adequate resuscitation or rising serum creatinine. Discontinuing the infusion was likewise at the discretion of the attending physician. Usual end-points were resolution of serum creatinine elevation, normalization of physiology, and restoration of urine output. In general, we dose fenoldopam infusion at 0.09 μg/kg/min and wean off when there is resolution of AKI as evidenced by SCr returning to baseline.

Data were obtained from the COLLECTOR database and the patient's electronic medical record. The COLLECTOR database is maintained by the United States Army Institute of Surgical Research (USAISR) and contains detailed demographic, laboratory, and treatment information on all patients admitted to the BICU. A retrospective analysis was conducted of patients who received low-dose fenoldopam and were admitted to the BICU. Patients were excluded if their mechanism was other than thermal burn or if they received any form of renal replacement therapy.

Demographic, laboratory, and physiologic data were obtained and outcomes determined. Data compiled for analysis included demographic data, admission vital signs, admission laboratory tests, injury severity scale (ISS) scores, and mortality. Vital signs and laboratory tests taken on admission were systolic blood pressure (SBP), diastolic blood pressure (DBP), pulse, temperature (ºF), blood urea nitrogen (BUN), and serum creatinine (SCr). Recorded vital signs and compiled laboratory results were the earliest available after admission. Additionally, SCr, SBP, DBP, vasoactive medication use, intravenous fluid (IVF) requirement, and urine output (UOP) were measured serially prior to initiation of fenoldopam. The only vasoactive medications utilized included norepinephrine, dobutamine, and vasopressin. The dose of vasoactive agents is expressed as the *modified inotropic score*, a dimensionless variable calculated as (dobutamine dose × 1) + (norepinephrine dose × 100) + (vasopressin dose × 100), wherein dobutamine and norepinephrine doses are expressed as μg/kg/min and vasopressin dose is expressed as units/min [30-33]. A dose-response relationship between vasoactive medication dose and mean arterial pressure (MAP) was used as another surrogate measure for the degree of hemodynamic impairment. This relationship is expressed as the *vasopressor dependency index*, which is the ratio of *modified inotropic score *to MAP; the higher the index, the more dependent the patient is on vasoactive medications [34].

A chart review was performed to determine which patients had a possible diagnosis of concurrent sepsis. Patients were also classified by the AKI Network (AKIN) scoring criteria [27]. AKIN stage was determined at the time of initiation of fenoldopam using the lowest SCr during admission as baseline.

Individual ISS scores were calculated from patient medical records according to published guidelines [35,36].

Microsoft Office Excel 2003 (Microsoft Corp, Redmond, WA) was used for database construction. Serial measurements were compared with one-way analysis of variance (ANOVA) with repeated measures and Wilcoxson signed rank test. Categorical variables were described with chi-square analysis using SPSS 16.0 (Cary, NC). Variables are expressed as median with intraquartile range or mean and standard deviation, and statistical significance was set at a *p *value of less than 0.05.

## Results

**Figure 1:**
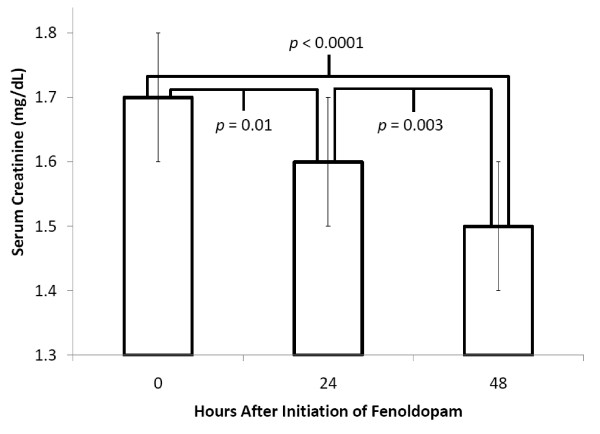
**Serum creatinine (SCr) over time following the initiation of fenoldopam**. SCr improved over the first 24 hours and continued to improve at 48 hours after fenoldopam initiation. Data presented as mean with standard error.

**Figure 2:**
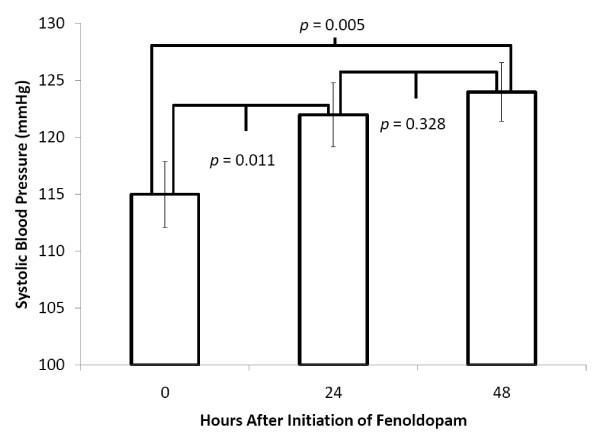
**Systolic blood pressure over time following the initiation of fenoldopam**. Systolic blood pressure increased during the initial 24 hours after fenoldopam initiation. This increase was maintained at 48 hours. Data are presented as mean with standard error.

Between November 2005 and September 2008, 758 patients were admitted to the BICU. Of these, 77 patients (10%) were treated with low-dose fenoldopam. Patient demographics are displayed in Table 1. Patients were stratified by AKIN criteria and their mortality rates are presented in Table 2. 5/77 (6%) received fenoldopam within 24 hours of admission and 24/77 (31%) received fenoldopam within 48 hours of admission. SCr improved over the first 24 hours and continued to improve through 48 hours (Figure 1). SBP increased in the first 24 hours and was sustained through 48 hours after initiation of fenoldopam (Figure 2). Mean arterial pressure increased over the first 24 hours and this increase was sustained at 48 hours after initiation of fenoldopam (76 ± 16 vs. 81 ± 15 vs. 81 ± 15; *p *< 0.05).

**Table 1:** Patient Demographics

n = 77	Mean (SE)	Median (IQR)
Age (yr)	42 (2)	37 (24-57)
ISS	28 (2)	25 (16-34)
TBSA (%)	42 (3)	40 (23-58)
Vent Days	35 (4)	21 (7-55)
ICU Days	56 (6)	45 (18-72)
Hospital Days	79 (8)	63 (31-100)
Inhalation Injury	28%	
Concurrent Sepsis	60%	

**Table 2:** AKI and Mortality by Stage

	n (%)	Mortality n(%)
All	77 (100%)	29 (38%)
AKI	76 (99%)	29 (38%)
Stage 1	20 (26%)	9 (45%)
Stage 2	14 (18%)	3 (21%)
Stage 3	42 (55%)	17 (40%)

Patients stratified by the Acute Kidney Injury Network classification system

**Figure 3:**
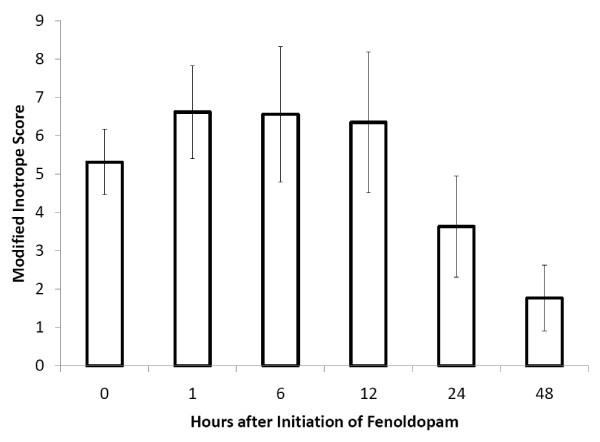
**Modified inotrope score over time following the initiation of fenoldopam**. The modified inotrope score decreased over 48 hours by one-way ANOVA with repeated measures. This change became significant at 24 hours. p = 0.0001 and p = 0.004 respectively. Data presented as mean with standard error.

**Figure 4:**
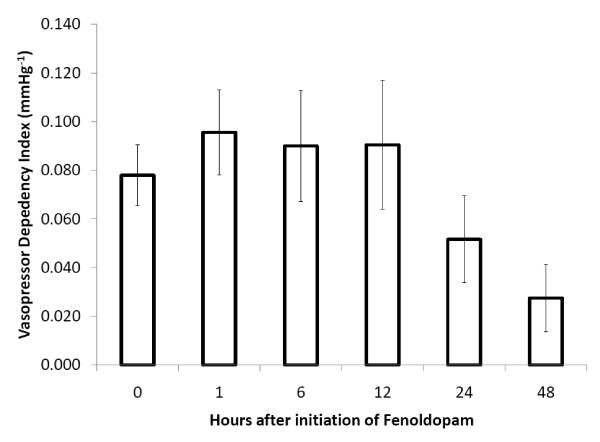
**Vasopressor dependency index over time following the initiation of fenoldopam**. The vasopressor dependency index decreased over 48 hours by one-way ANOVA with repeated measures. This change became significant at 48 hours. p = 0.0012 and p = 0.02 respectively. Data presented as mean with standard error.

Upon initiation of fenoldopam in our study population, 9% (7/77) were on vasopressin alone, 3% (2/77) were on norepinephrine alone, 6% (5/77) were on dobutamine alone, and 8% (6/77) were on a combination of the three. The *modified inotrope score *and the *vasopressor dependency index *both decreased over 48 hours by one-way ANOVA with repeated measures (*p *< 0.0001, *p *= 0.0012) (Figures 3 and 4).

Scr improved at 24 hours and continued to improve at 48 hours in all AKIN stages when patients were stratified by stage (Table 3). UOP increased after initiation of fenoldopam without an associated increase in IVF. UOP was significantly increased in the 12 hours after initiation of fenoldopam overall and in stratified AKIN stages 2 and 3 patients (Table 4). IVF was not different pre- and post-fenoldopam initiation overall and when stratified by stage (Table 5). Overall mortality for our cohort was 38% (29/77).

**Table 3:** SCr (mg/dL) by AKIN Stage and Time

AKIN Stage	Pre-Fenoldopam		24 Hours		48 Hours	
	**Mean (SE)**	**Median (IQR)**	**Mean (SE)**	**Median (IQR)**	**Mean (SE)**	**Median (IQR)**
ALL	1.7 (0.1)	1.7 (1.4-1.9)	1.6 (0.1)	1.4 (1.2-1.9)	1.5 (0.1)	1.3 (1.0-1.6)
1	1.5 (0.1)	1.5 (1.3-1.7)	1.3 (0.1)	1.3 (1.0-1.5)	1.2 (0.1)	1.1 (0.9-1.4)
2	1.7 (0.1)	1.9 (1.7-1.9)	1.8 (0.3)	1.5 (1.3-1.9)	1.3 (0.1)	1.3 (1.0-1.7)
3	1.8 (0.1)	1.7 (1.4-2.0)	1.8 (0.2)	1.5 (1.2-2.1)	1.7 (0.2)	1.4 (1.1-2.1)

*p *< 0.05 for all time-time interactions

**Table 4:** UOP (mL/hr) by AKIN Stage and Time

AKIN Stage	12 Hours Pre-fenoldopam		12 Hours Post-fenoldopam		*p *value
	**Mean (SE)**	**Median (IQR)**	**Mean (SE)**	**Median (IQR)**	
ALL	79 (10)	54 (33-84)	93 (8)	82 (55-111)	0.000
1	68 (11)	54 (43-71)	76 (6)	78 (53-92)	0.133
2	46 (8)	37 (28-58)	72 (15)	57 (33-90)	0.009
3	94 (16)	59 (33-108)	107 (13)	87 (64-116)	0.009

**Table 5:** IVF (mL/hr) by AKIN Stage and Time

AKIN Stage	12 Hours Pre-fenoldopam		12 Hours Post-fenoldopam		*p *value
	**Mean (SE)**	**Median (IQR)**	**Mean (SE)**	**Median (IQR)**	
ALL	375 (25)	314 (241-431)	365 (27)	324 (232-457)	0.782
1	439 (38)	334 (271-568)	406 (86)	327 (201-398)	0.751
2	370 (56)	278 (231-421)	366 (59)	341 (157-494)	0.964
3	351 (30)	305 (254-385)	352 (22)	317 (258-453)	0.971

## Discussion

Our retrospective study of low-dose fenoldopam to treat AKI in critically ill burn patients demonstrated an improvement in multiple markers of renal function within the first 48 hours of therapy. These positive changes included a decrease in SCr, an increase in UOP, and decrease in the modified inotrope score and vasopressor dependency index with essentially no change in IVF administration. The use of fenoldopam in this study appeared safe, without the hypotensive complications that have historically been associated with this medication. To our knowledge, this study is the first to describe the use of fenoldopam for AKI in the burn population.

Improvement in renal function occurred in the group as a whole and within each subgroup when they were broken down by AKI severity. This result suggests that patients with all stages of AKI may benefit from the increased RBF afforded by fenoldopam infusion. The improvement in SCr was 26% for those with AKIN stage 1, 31% for AKIN stage 2, and 18% for AKIN stage 3. The smaller improvement in AKIN stage 1 compared to stage 2 likely reflects a lower specificity for AKI by using the new staging criteria for stage 1 so that some patients without true AKI may have been included in the study [27,37,38]. Additionally, the relatively low initial SCr in patients with AKIN stage 1 limited the percentage of improvement that could be demonstrated in this group. Though still demonstrating benefit, the smallest percentage of improvement was seen in those patients with AKIN stage 3. The limited response in this group probably reflects more severe underlying damage that has moved beyond mere ischemia and is no longer corrected with renal vasodilation.

Volume regulation is accepted as a critical aspect of resuscitation of the burn patient. UOP is generally utilized as one of the most consistent physiologic indicators of adequate renal perfusion and resuscitation in the burn population. Conversely, oliguria is generally accepted as a sign of inadequate renal perfusion and resuscitation. Any treatment strategy able to improve UOP while minimizing undesired effects deserves our consideration. Our use of fenoldopam was associated with a 50% increase in UOP in the first 12 hours after initiation. This improvement was seen in the group as a whole, as well as in AKIN stage 2 and stage 3 patients. While not achieving statistical significance, there was also a trend towards increasing urine output in patients classified as AKIN stage 1. The observed improvement in urine output can be attributed to fenoldopam based upon two separate facts: no significant change in the volume of intravenous fluid infused and a decrease in vasopressor requirements in the 48 hours following initiation of fenoldopam.

Given the mechanism of fenoldopam, early administration of the medication in the treatment and prevention of AKI may be beneficial. During the early phases of acute tubular necrosis (ATN), renal nerve stimulation, angiotensin II, thromboxane-A2, and endothelin lead to vasoconstriction [39,38-43]. RBF is dependent on renal vascular resistance (RVR) and systemic vascular resistance (SVR), such that if RVR increases relative to the SVR, RBF will decrease [44] RBF has been shown to decrease during the first 24 hours of ATN, and the kidney loses its ability to autoregulate, such that during the first 12 to 24 hours of AKI, there is a direct relationship between renal perfusion pressure (RPP) and RBF [45-47]. More specifically, it is the renal outer medullary blood flow that is decreased, at least partially, secondary to cellular detachment and luminal occlusion [48].

Prior work has demonstrated that in post-ischemic AKI, nitric oxide synthase (NOS) activity is maximal at baseline and cannot be increased further by other stimuli of NOS activity [4]. Therefore, attempting to increase RBF via nitric oxide-dependent vasodilators may prove unsuccessful [4,49]. Given fenoldopam's nitric oxide independent mechanism of action and its preferential corticomedullary blood flow augmentation and ability to increase oxygenation, it is conceivable that it provides additional RBF benefits over other commonly used vasodilators such as nitroprusside [50-52].

Several authors have pointed out potential pitfalls with the use of fenoldopam, including the potential for ischemia reperfusion injury secondary to the increased production of reactive oxygen species [49,53,54]. Additionally, given the risk of hypotension inherent with the use of fenoldopam, subsequent renal hypoperfusion is a conceivable side effect [55]. However, our patients experienced an actual improvement in their UOP and a decrease in their SCr, arguing against the presence of renal ischemia reperfusion injury. Furthermore, there was not an increased incidence of hypotension or in the patient's fluid requirements, and there was a decrease in the use of vasoactive medications. Moreover, Kien et al. demonstrated that despite a MAP of < 60 mm Hg, both cortical and medullary blood flow were increased by 30% and 40%, respectively, with the use of fenoldopam, suggesting that fenoldopam prevents the redistribution of blood away from these critical regions during conditions that can lead to AKI [51].

Our study has the inherent weaknesses of all retrospective studies. We were limited by the data available as well as the lack of an appropriate control group to compare our findings against. The study was not adequately powered to determine more clinically significant end points such as rate of dialysis requirement, hospital or ICU length of stay, differences based on etiology of renal dysfunction, or mortality. We also excluded patients who received renal replacement therapy. Renal replacement therapy artificially lowers the serum creatinine and alters the body's physiology such that gross measures of resuscitation and renal physiology would not be applicable (i.e. IVF, UOP, SCr). This limited the number of patients available for our study. Additionally, although most patients likely had ischemic ATN as the etiology of their AKI, there is potential that other etiologies such as nephrotoxic ATN could have contributed to their AKI.

## Conclusions

We have demonstrated that low-dose fenoldopam was associated with an improvement in renal function and UOP not related to additional volume resuscitation. Additionally, there is no evidence, in our population, that low-dose fenoldopam was associated with adverse hemodynamic effects. Given the high morbidity and mortality associated with AKI in this population, even marginal improvements in SCr and UOP may translate into more meaningful outcomes. It is clear that a safe, well-tolerated modality is needed to prevent and treat AKI. A randomized controlled trial is required to see whether low-dose fenoldopam can serve as that modality in critically ill burn patients with AKI.

The key messages resulting from our retrospective study are as follows:

- Fenoldopam is associated with improved hemodynamics and increased UOP.

- Improvements are not associated with increased resuscitation or vasoactive medication requirements.

- Fenoldopam appears to be a safe adjunct in the treatment of AKI in burned patients.

## Abbreviations

Abbreviations used in the manuscript include: ADH:anti-diuretic hormone; AKI:acute kidney injury; AKIN:Acute Kidney Injury Network; ATN:acute tubular necrosis; BICU:burn intensive care unit; BUN:blood urea nitrogen; DBP:diastolic blood pressure; GFR:glomerular filtration rate; ICU:intensive care unit; IQR:interquartile range; IVF:intravenous fluid; MAP:mean arterial pressure; RBF:renal blood flow; RVR:renal vascular resistance; SBP:systolic blood pressure; SCr:serum creatinine; SD:standard deviation; TBSA:total body surface are; UOP:urine output.

## Competing interests

All authors declare that we have no competing interests.

## Authors' contributions

JWS was involved with design, data acquisition, analysis, and manuscript drafting. KKC was involved with study conception, design, data analysis, manuscript drafting, and editing. EMR and CEW were involved with study conception and manuscript editing. CLC and MAT were involved with data analysis and manuscript drafting. MOH were involved with data acquisition. JAJ was involved with statistical analysis and manuscript drafting. LHB was involved with editing and final approval of the manuscript. SEW was involved with study design, manuscript editing, and supervision of the research group. All authors read and approved the final manuscript.

## Authors' information

JWS and MOH are general surgery residents.

KKC (medical intensivist) is the Medical Director of the burn ICU.

EMR is a burn/trauma surgeon and also the current director of the US Army Burn Center.

CEW is a burn/trauma surgeon.

CLC and MAT are nephrologists.

JAJ is a statistician.

LHB is the commander of the US Army Institute of Surgical Research and a trauma surgeon.

SEW (burn surgeon) is the former Burn Director of the US Army Burn Center and current director of research. He is also the editor-in-chief of Burns.

## Pre-publication history

The pre-publication history for this paper can be accessed here:

http://www.biomedcentral.com/1471-2253/10/9/prepub
